# Research hotspots and frontiers of essential tremor from 2013 to 2023: a visualization analysis based on CiteSpace

**DOI:** 10.3389/fnagi.2024.1380851

**Published:** 2024-07-23

**Authors:** Linlin Zhang, Shifang Cui, Xiaoming Xi, Hongyan Bi, Bin Huang

**Affiliations:** ^1^Nantong Fourth People’s Hospital, Nantong, China; ^2^Heilongjiang University of Chinese Medicine, Harbin, China; ^3^Beijing Rehabilitation Hospital, Capital Medical University, Beijing, China; ^4^Shandong University of Traditional Chinese Medicine Affiliated Hospital, Jinan, China

**Keywords:** essential tremor, Citespace, visualization, emerging trends, knowledge graph

## Abstract

**Background:**

ET, one of the most prevalent neurological disorders, presents a significant challenge in terms of disability. Despite the growing focus on ET in recent years, comprehensive bibliometric analysis has been lacking.

**Methods:**

This study delves into essential tremor research covering the period from 2013 to 2023, utilizing the Web of Science (WOS) database. Employing CiteSpace for quantitative analysis, it examines an array of metrics including annual publication trends, contributions from countries and institutions, authorship patterns, key terminologies, and patterns of reference co-citation. The primary objective is to use CiteSpace for a detailed visual exploration of the literature over the last decade, pinpointing the evolving landscape and key areas of focus in essential tremor research, and thus providing a foundation for future investigative endeavors.

**Results:**

There were 2,224 literary works included in all. The amount of published works has been steadily rising in recent years. Of them, the majority originate from the United States, Louis, Elan D. is the publisher of the most publications (161 articles), and Movement Disorders is the journal that receives the most citations. The key words contribution and co-cited literatures suggest that the main research hotspots in recent years are the physiological and pathological mechanism of essential tremor, the determination of optimal targets for deep brain stimulation (DBS) and surgery transcranial magnetic resonance-guided focused ultrasound (MRgFUS) in the surgical management of essential tremor and the innovative research of botulinum toxin administration method.

## 1 Introduction

Essential tremor is a distinct tremor syndrome marked by bilateral upper limb action tremors persisting for a minimum of 3 years, either accompanied or unaccompanied by tremors in other regions (e.g., head, voice, or lower limbs), yet devoid of other neurological manifestations like muscle tone abnormalities, ataxia, or Parkinson’s syndrome (Pandey, 2022). The worldwide prevalence of essential tremor stands at 0.9%, with the incidence progressively escalating with age, surging by 74% per decade and peaking at 5% among individuals aged 65 and above (Louis and McCreary, 2021).

In terms of treatment, pharmacotherapy, primarily with Propranolol and Primidone, has long been the cornerstone of ET management, although only about half of the patient population responds effectively to these medications (Shanker, 2019; Castonguay et al., 2022; Frei and Truong, 2022). Alternative treatments such as Topiramate (Chang et al., 2015; Bruno et al., 2017) and benzodiazepines (Bruno et al., 2020; Miyamoto and Ito, 2023) serve as secondary options but carry the risk of exacerbating cognitive issues in ET patients. Surgical interventions, namely deep brain stimulation and focused ultrasound thalamotomy, have emerged as vital options for those resistant to drug therapy, demonstrating promising results in tremor control (Elble et al., 2018; Ferreira et al., 2019; Hopfner and Deuschl, 2020). Concurrently, ongoing research is seeking to identify more efficacious treatment targets (Fan et al., 2022; Kondapavulur et al., 2022). Additionally, exercise therapies, including resistance training (Kavanagh et al., 2016; Bryant et al., 2018) and yoga (Vance et al., 2019), have shown potential in the amelioration of tremors, though a comprehensive approach to exercise prescription in ET treatment is still in its early stages.

In recent years, research on essential tremor has progressed, leading to a steady rise in the number of related research publications. However, literature that comprehensively explores, analyzes, and summarizes the advancements in this field is currently lacking. Bibliometrics, a vital academic discipline, is dedicated to evaluating the quantitative characteristics, trends, and academic influence of scientific literature. Researchers utilize bibliographic retrieval systems and metric analyses to gain a comprehensive understanding of publications from various perspectives, enabling the assessment of their historical, current, and future significance, as well as their quantity and quality (Montazeri et al., 2023). This study offers a multifaceted examination of ET-related literature published in the Web of Science over the past decade utilizing the bibliometric tool CiteSpace. It involves an analysis of publication output by country and institution, along with co-authorship, co-citation, and co-occurrence analyses to elucidate the developmental trends in this field, identify current research focal points, and predict future research directions. Lastly, the findings are analyzed and synthesized to serve as a reference for future research pathways in ET.

## 2 Materials and methods

### 2.1 Data source and search strategy

**Figure 1:**
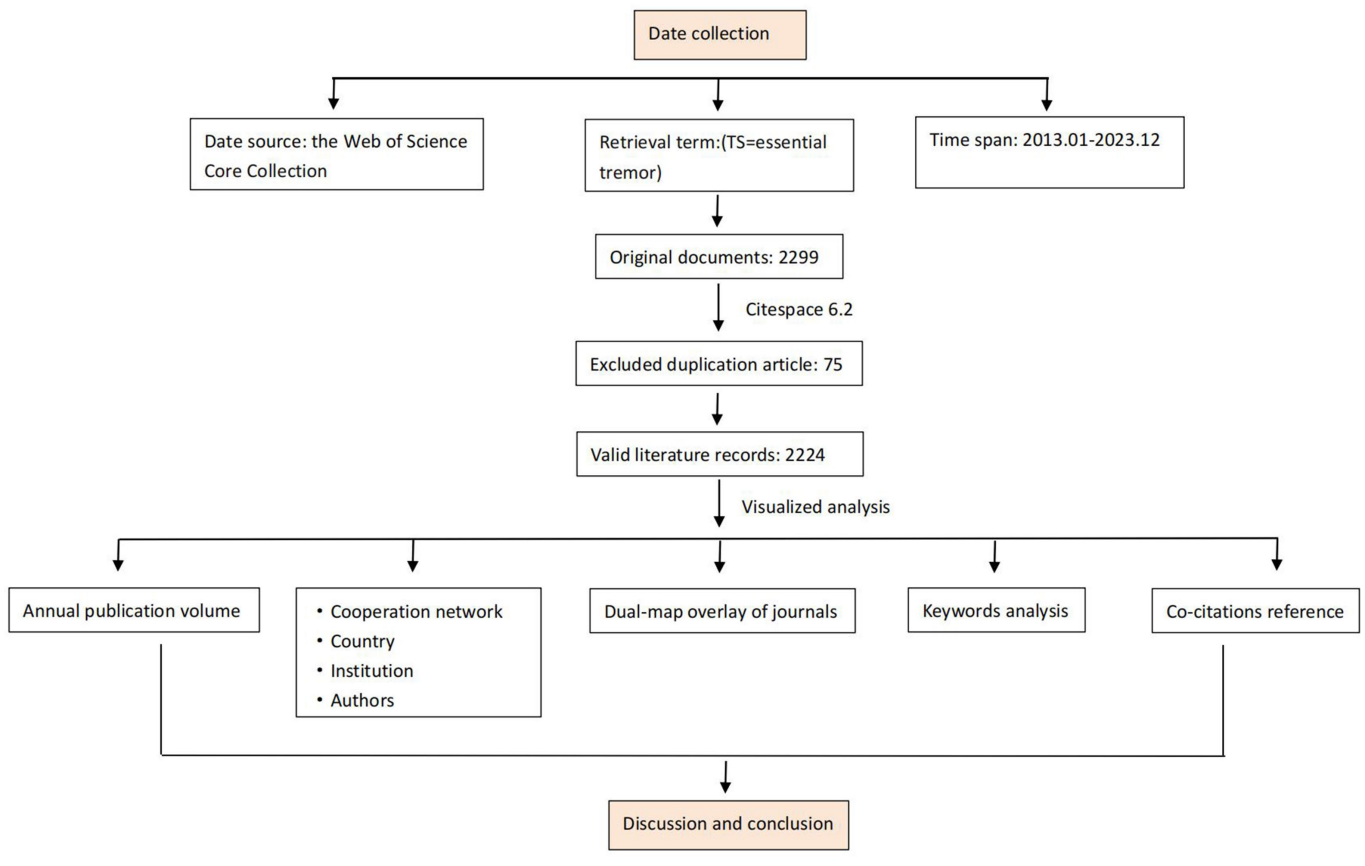
Illustrates the flow chart of the study design.

A comprehensive search for literature pertaining to Essential Tremor was conducted using the Web of Science Core Collection (WOSCC). Inclusion criteria include literature published from 2013 to 2023, peer-reviewed original articles on ET, encompassing both basic and clinical research, reviews on ET, with no language restrictions. And excluding unpublished articles, conference abstracts, conference proceedings, and duplicated publications. A total of 2,224 articles meeting the criteria were ultimately included (Figure 1).

### 2.2 The parameters for CiteSpace

CiteSpace is a Java-based scientometric software that generates visual maps to vividly display the development patterns, literature element structures, distributions, and other systematic content. It classifies and clusters information in research areas through bibliographic data, enabling a more scientific and intuitive analysis of the development history and trends of specific fields, and establishes co-linear knowledge networks.

Data analysis was conducted by importing 2,224 research papers into the pre-installed Citespace software designed for bibliometric analysis. Time slice was set to 1 year; the analysis period spanned from January 2013 to December 2023; the term source included all items, analyzed one node type at a time; all other settings were maintained at their default values.

## 3 Results

### 3.1 Analysis of publication volume

The past decade has witnessed a consistent upward trend in the volume of literature related to Essential Tremor (see Table 1). This trend can be segmented into three distinct phases: The initial phase (2013–2017) experienced a steady increase in annual publication volume. This was followed by a second phase (2017–2020), marked by a fluctuating rise in publication volume, there was a minor decline in 2019, followed by a peak in 2020 with 261 articles. Since 2021, during the third phase, there has been a gradual decrease in the number of publications.

**Table 1:** The annual number of global publications and citations from 2013 to 2023.

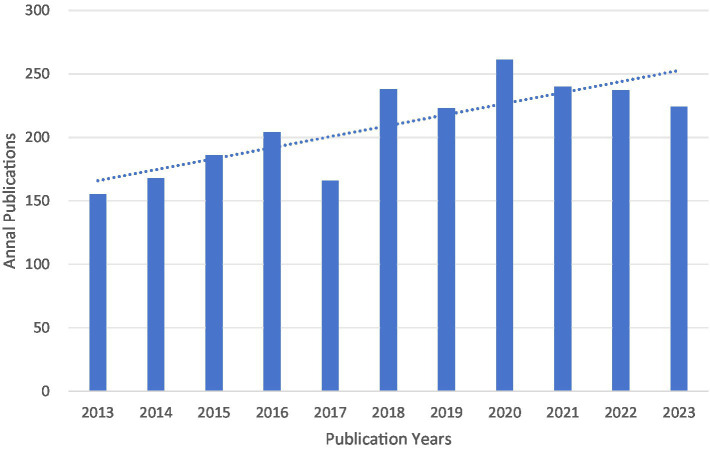

### 3.2 Country co-occurrence analysis

**Figure 2:**
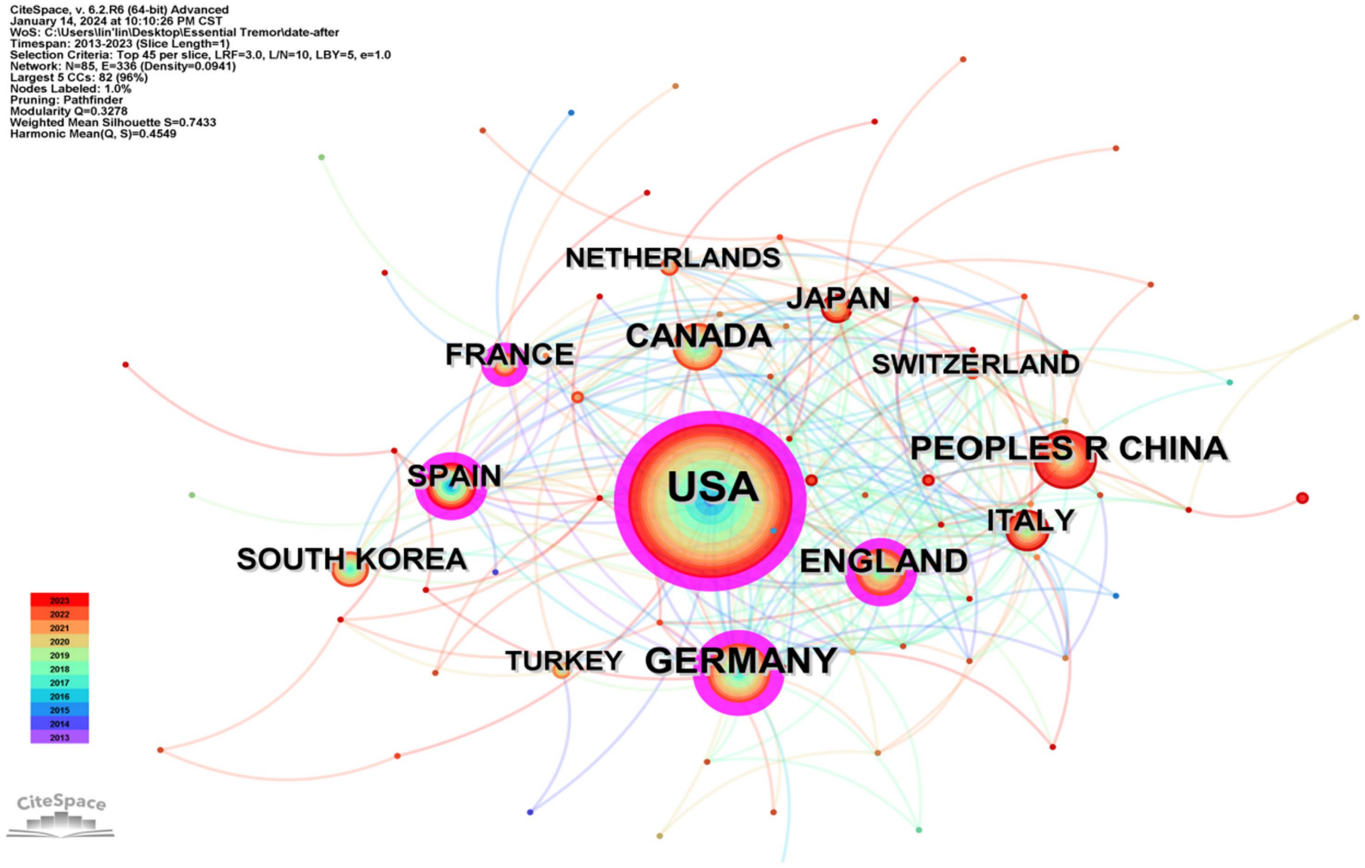
Research collaboration map between countries.

**Figure 3:**
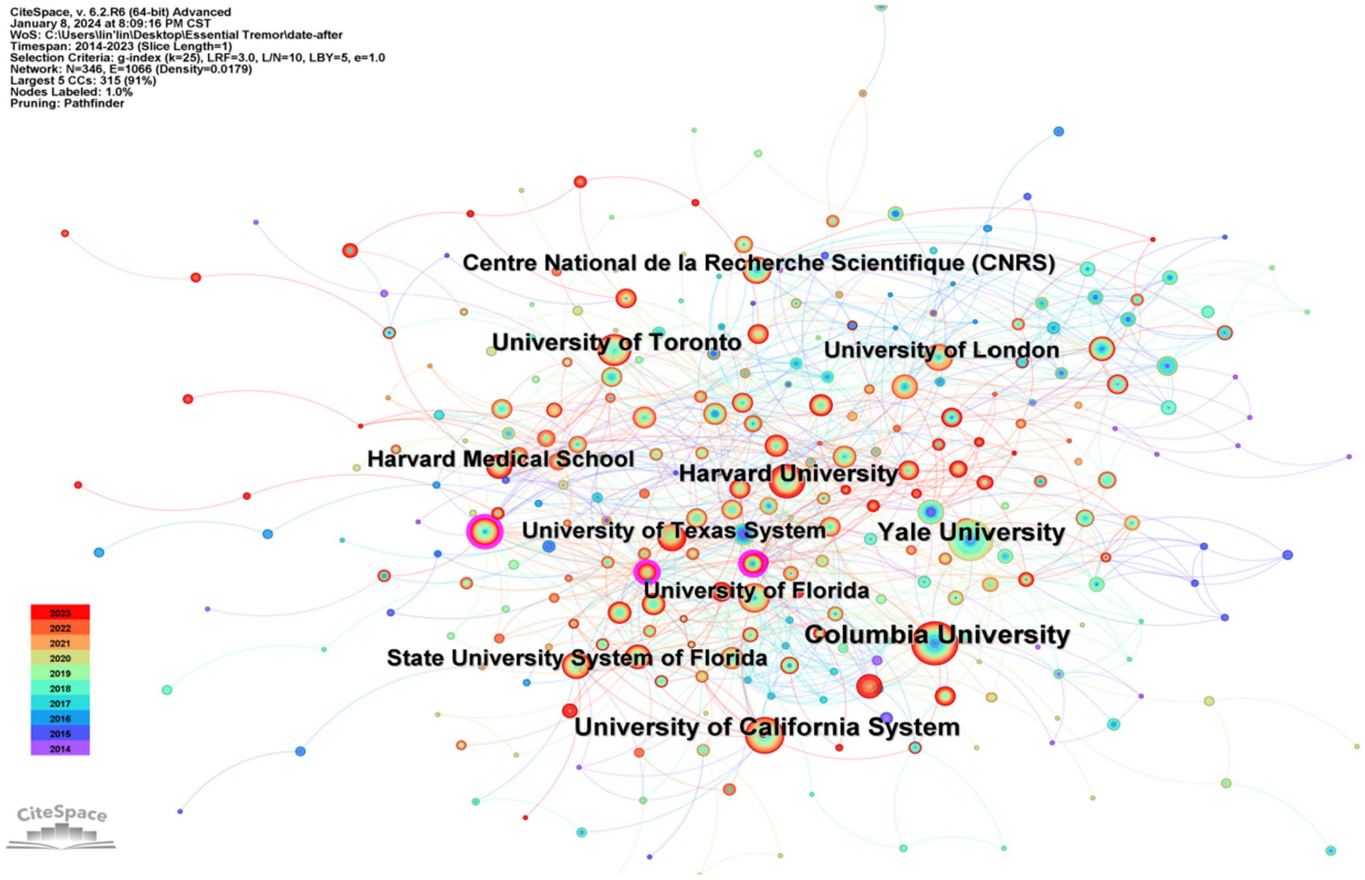
Research collaboration map between institutions.

Through visual analysis using countries and institutions as nodes, a network graph depicting country/institution collaboration was generated (Figures 2, 3). Eighty-five countries have contributed to research on Essential Tremor. The United States is at the forefront with 971 publications, surpassing the combined total of the next five leading countries. The purple circles outside the main circle represent betweenness centrality, a measure used to assess the likelihood of a node being on the shortest path within the network graph, indicative of the node’s significance in the graph (nodes with a betweenness centrality value greater than 0.1 are considered key). Among the top 10 countries in terms of publication volume, Germany boasts the highest betweenness centrality (0.33).

The five institutions leading in publication volume are Columbia University (133 publications), Yale University (109), University of California System (91), University of Toronto (88), and Harvard University (81), all hailing from the United States. However, the betweenness centrality for these institutions is below 0.1, suggesting a relative dispersion in research collaboration among them (Table 2).

**Table 2:** The top 10 institutes and countries in terms of publication count and centrality.

Country	Count	Centrality	Institute (Country)	Count	Centrality
United States	971	0.24	Columbia University (United States)	133	0.07
Germany	250	0.33	Yale University (United States)	109	0.02
China	233	0.03	University of California System (United States)	91	0.02
Canada	168	0.07	University of Toronto (United States)	88	0.01
England	159	0.2	Harvard University (United States)	81	0.01
Italy	143	0.15	University of London (England)	68	0.03
Spain	143	0.06	University of Texas System (United States)	66	0.02
Japan	104	0.09	State University System of Florida (United States)	66	0.03
France	98	0.15	Harvard Medical School (United States)	60	0.04
South Korea	98	0.01	Center National de la Recherche Scientifique (France)	58	0.01

Centrality: Centrality represents the importance of countries and institutions. The greater the Centrality, the more connections countries and institutions have with others, indicating a central position among them.

Annotation: Within the diagram, the varying sizes of the dots reflect the publication output of each institution; a larger dot signifies a higher number of publications. The connecting lines between these dots symbolize the collaborative ties between institutions, where thicker lines represent more extensive collaboration. Surrounding these dots are purple circles, indicative of betweenness centrality. The thickness of these purple rings correlates with the level of centrality, with a thicker ring denoting a higher degree of centrality.

### 3.3 Analysis of the authors’ collaboration network map

**Figure 4:**
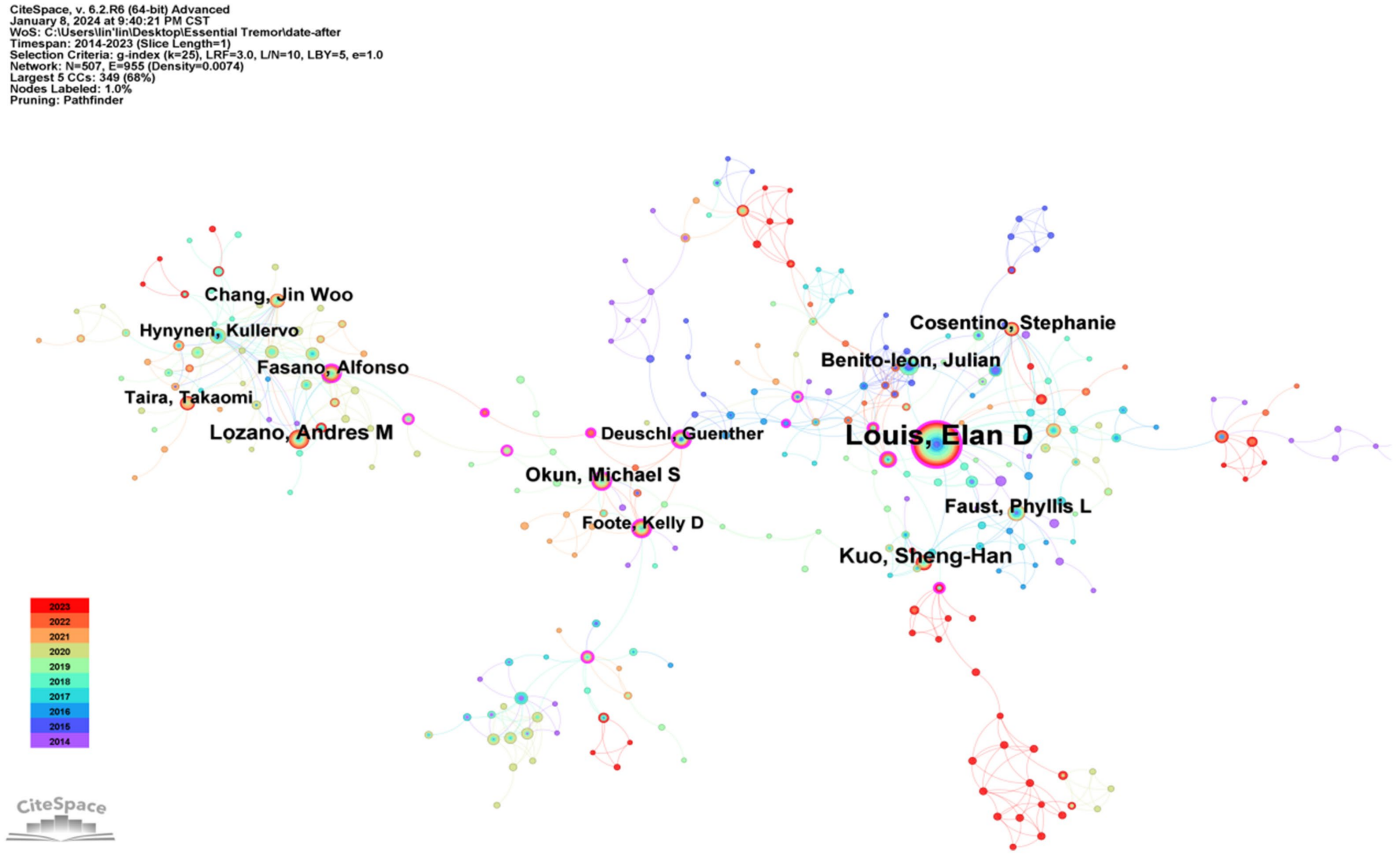
Author co-occurrence.

Employing “authors” as the focal nodes, a comprehensive visual analysis produced an intricate network map showcasing author collaborations, encompassing 507 nodes and 955 connecting lines (refer to Figure 4; Table 3). Over the past decade, the most distinguished author in Essential Tremor research has been Louis, Elan D from the University of Texas Southwestern Medical Center, contributing an impressive 161 publications and also achieving the highest betweenness centrality score of 0.25. This highlights Louis, Elan D’s extensive collaborations with other leading authors in the field, including Kuo, Sheng-Han (39 publications), Faust, Phyllis L (29 publications), and Cosentino, Stephanie (27 publications), who rank second, fourth, sixth, and seventh, respectively, in terms of publication volume. Such collaborations underscore Louis, Elan D’s pivotal role and substantial influence in Essential Tremor research. Notably, the third most prolific author, Lozano, Andres M (35 publications), maintains close collaborative relationships with the ninth and tenth-ranked authors, Fasano, Alfonso (24 publications), and Hynynen, Kullervo (20 publications), indicating the formation of a robust international academic coalition centered around Louis, Elan D and Lozano, Andres M.

**Table 3:** The top 10 authors and their organizations.

Author	Institute, Country	Count	Centrality
Louis, Elan D	University of Texas Southwestern Medical Center, United States	161	0.25
Kuo, Sheng-Han	Columbia University, United States	39	0.07
Lozano, Andres M	University Toronto, Canada	35	0.08
Benito-Leon, Julian	Complutense University of Madrid, Spain	29	0.05
Okun, Michael S	University Florida, United States	29	0.15
Faust, Phyllis L	Columbia University, United States	27	0.04
Cosentino, Stephanie	Yale University, United States	27	0.01
Chang, Jin Woo	Yonsei Univ, South Korea	25	0.09
Fasano, Alfonso	University Toronto, Canada	24	0.13
Hynynen, Kullervo	Sunnybrook Res Inst, Canada	20	0.01

Centrality represents the importance of authors. The greater the Centrality, the more connections the author has with other authors, indicating a central position among them.

### 3.4 Advanced visual analysis of journal dual map overlays

**Figure 5:**
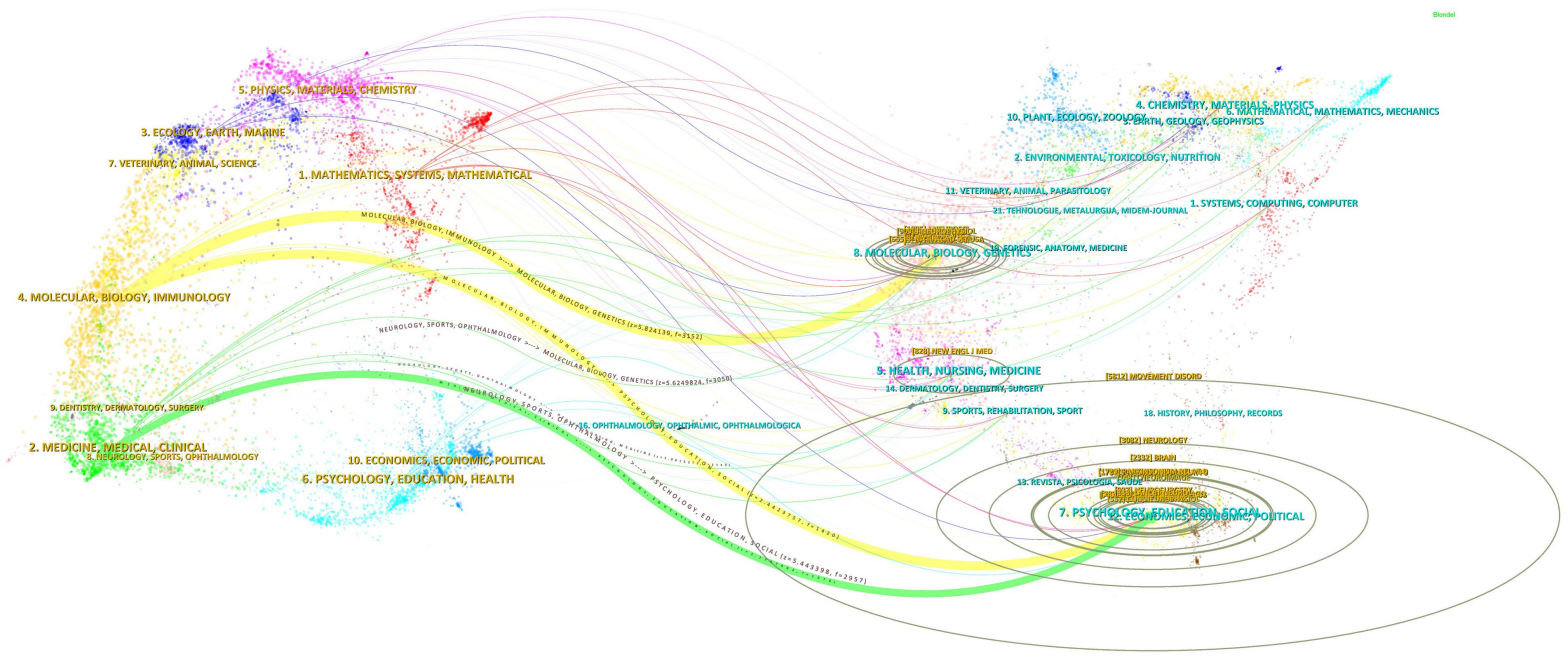
Journal dual map overlays.

The innovative dual map overlay functionality offers a detailed visual representation of the knowledge exchange between cited and citing articles, as showcased in Figure 5. The collection of citing journals described on the left side of the image represents the forefront of essential tremor research field. Conversely, the right side is grounded in the disciplines of the cited literature, establishing the foundational research landscape in the essential tremor field. The depicted curves elegantly trace the routes of citation paths.

A noteworthy highlight is the journal ‘Movement Disorders’ (Impact Factor = 9.698), which emerges as the most frequently cited publication in this domain. The analysis identifies three principal citation trajectories. The orange trajectory reveals a significant influence of journals in the Molecular/Biological/Immunology fields by works published in Molecular/Bio/Genetics (*z* = 5.824, *f* = 3,152) and Psychology/Education/Sociology (*z* = 2.443, *f* = 1,420). Similarly, the green trajectory underscores how publications in the Medicine/Medical/Clinical spheres are substantially shaped by insights from the Psychology/Education/Sociology (*z* = 1.771, f = 1,076) disciplines. The Journal Dual Map Overlays prediction of journal maps suggests that the hotspots and trends in essential tremor research will converge in the molecular/biological/pharmacological domain.

Annotation: In the figure, each ellipse symbolizes the volume of publications associated with a specific journal. The length of an ellipse’s horizontal axis represents the number of authors involved, whereas its vertical axis length indicates the quantity of papers published by the journal. Z: The Z-score is a statistical metric employed to ascertain the relative position of a specific data point within a dataset. F: Frequency of citations.

### 3.5 Keyword co-occurrence analysis

**Figure 6:**
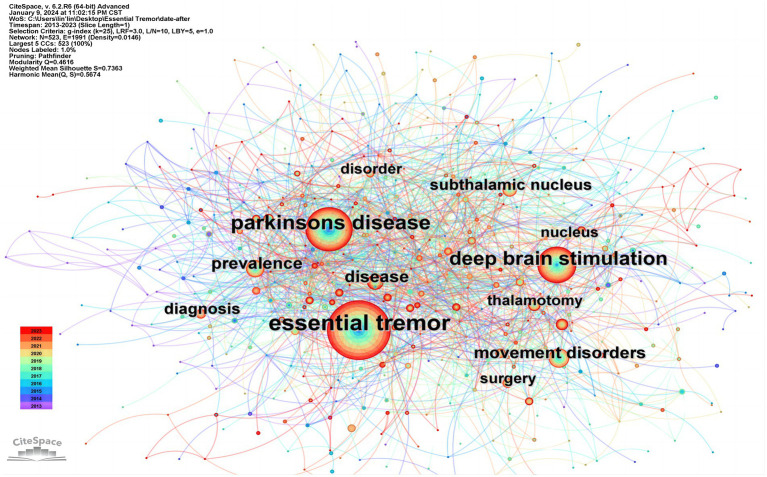
A network map of co-occurring keywords.

The network map showcasing co-occurring keywords vividly illustrates the principal research areas (as seen in Figure 6; Table 4), This analysis principally revolves around the frequency of occurrence of these keywords, Moving beyond central thematic terms, the most recurrent keywords over the past decade have been “Parkinson’s disease,” “deep brain stimulation,” “movement disorders,” “prevalence,” “disease,” “subthalamic nucleus,” “diagnosis,” “thalamotomy,” and “surgery”.

**Table 4:** The top 10 most frequent keywords.

Rank	Keywords	Count	Centrality
1	Essential tremor	1,366	0
2	Parkinsons disease	775	0
3	Deep brain stimulation	542	0.02
4	Movement disorders	206	0.03
5	Prevalence	195	0.01
6	Disease	186	0.02
7	Subthalamic nucleus	149	0.01
8	Diagnosis	131	0.01
9	Thalamotomy	117	0.03
10	Surgery	114	0.03

### 3.6 Keyword clustering analysis

Clustering map of co-occurring keywords presents a clustering map of co-occurring keywords, visually depicting the interconnectedness and thematic grouping of these research areas.

**Figure 7:**
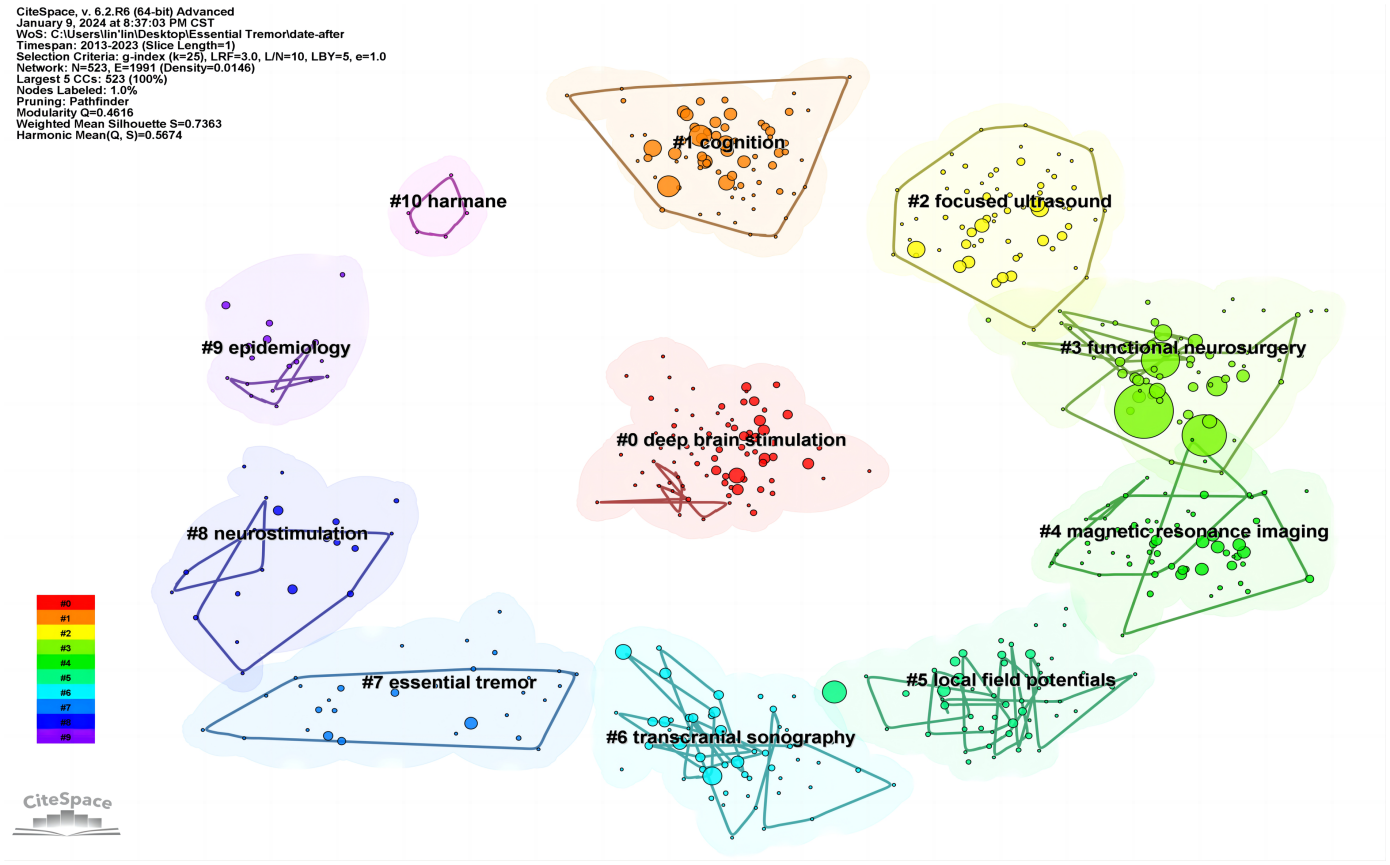
Clustering map of co-occurring keywords.

The keyword clustering analysis reveals a Q value of 0.4616, surpassing the threshold of 0.3, and an S value of 0.7363, exceeding 0.7. These values collectively suggest a high level of trustworthiness in the clustering outcomes. Specifically, a Q value above 0.3 indicates a pronounced community structure within the clustering, while an S value greater than 0.7 reflects a robust credibility in the results. The analysis successfully identified 11 distinct clustering labels, each representing a unique subfield in the study of essential tremor. These labels are further grouped into thematic classes: #0, #2, #3, #6, and #8 form the first class, focusing primarily on the treatment research of essential tremor; #4, #5, #7, and #10 constitute the second class, centered on exploring the mechanisms of essential tremor; and #1 and #9 make up the third class, dedicated to investigating the symptoms and epidemiological aspects of essential tremor. These classifications are illustrated in Figure 7 and detailed in Table 5.

**Table 5:** List of keywords cluster labels of postural control studies in ET.

Cluster-ID	Size	Silhouette	Cluster name	Cluster labels (LLR)
#0	77	0.696	Deep brain stimulation	Deep brain stimulation; gene; mutations; genetics; purkinje cell
#1	71	0.764	Cognition	Deep brain stimulation; cognition; clinical; depression; dementia
#2	66	0.691	Focused ultrasound	Focused ultrasound; thalamotomy; mr-guided focused ultrasound; mrgfus; thermal ablation
#3	63	0.742	Functional neurosurgery	Deep brain stimulation; functional neurosurgery; tractography; zona incerta; ventral intermediate nucleus
#4	62	0.692	Magnetic resonance imaging	Magnetic resonance imaging; cortical thickness; motor cortex; connectivity; voxel-based morphometry
#5	54	0.617	Local field potentials	Local field potentials; synchrony; oscillations; parkinsonian tremor; neuronal oscillations
#6	53	0.8	Transcranial sonography	Transcranial sonography; substantia nigra; datscan; deep brain stimulation; multiple system atrophy
#7	30	0.869	Essential tremor	Essential tremor; validation; neurons; deterministic tractography; probabilistic tractography
#8	20	0.902	Neurostimulation	Deep brain stimulation (dbs); neurostimulation; gait; intention tremor; kinematic analysis
#9	17	0.889	Epidemiology	Epidemiology; population-based study; elderly; death certificates; premotor symptoms
#10	5	0.99	Harmane	Harmane; toxicant; toxin; beta-carboline alkaloid; epidemiology

*LLR is Log-Likelihood Ratio.

### 3.7 Analysis of keyword bursts

**Figure 8:**
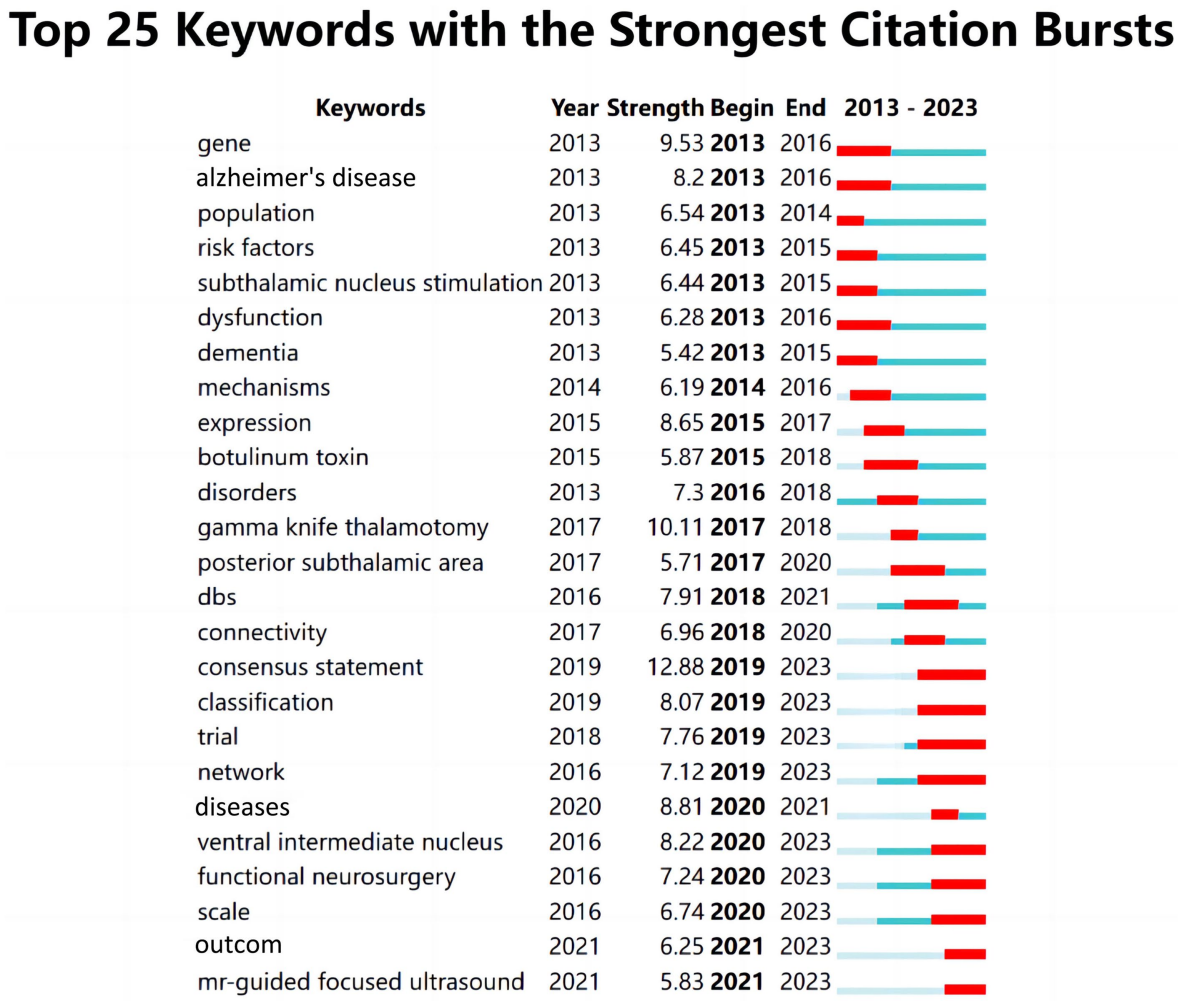
The top keywords with the strongest citation bursts.

Research on keyword citation bursts provides a clear reflection of the predominant research topics within a field’s literature over a specific period, as depicted in Figure 8. Analyzing the timeframe from 2013 to 2016, the prominent keywords that surged were gene, Alzheimer’s disease, population, risk factors, subthalamic nucleus, stimulation, dysfunction, dementia, and mechanism. This highlights a focus on functional disorders and epidemiological characteristics of essential tremor. From 2017 to 2019, the emerging keywords shifted to ‘expression, botulinum toxin, disorders, gamma knife thalamotomy, posterior subthalamic area, DBS, connectivity,’ signaling a global research interest in the mechanisms and surgical interventions for essential tremor. Botulinum toxin, a treatment option for essential tremor since the 1990s (Demiryurek, 2021; Mittal and Pandey, 2022), had not been widely adopted due to side effects such as muscle weakness following injection. However, recent advancements in injection techniques have revitalized interest in botulinum toxin as a potential treatment (Mittal et al., 2020). “Gamma knife thalamotomy and dbs” is the surgical treatment for idiopathic tremor (Ferreira et al., 2019; Horisawa et al., 2023)”. Posterior subthalamic area” is one of the common targets for DBS (Ferreira et al., 2019).

The period from 2020 to 2023 marks the third phase, with key terms including consensus statement, classification, trial, network, disease, ventral intermediate nucleus, functional neurosurgery, scale, outcome, and MR-guided focused ultrasound. This era witnessed the maturation of research in essential tremor, notably marked by the development of consensus statements. Comparative studies of different surgical techniques also increased. Fan et al. (2022) conducted a review of recent clinical research on the ventral intermediate nucleus (VIM) and posterior subthalamic area (PSA) deep brain stimulation (DBS). Their findings suggest that while both PSA-DBS and VIM-DBS are effective in reducing tremors, PSA-DBS is more effective and safer over a 12–24 month period. Furthermore, MR-guided focused ultrasound (MRgFUS) has gained significant attention in recent years. This incision-free surgical technique, which utilizes focused ultrasound for thalamotomy, poses fewer risks and offers substantial improvement in tremor symptoms (Gallay et al., 2020; Wüllner, 2020). However, it is associated with considerable side effects, as reports indicate that up to 15% of patients may experience permanent deficits such as sensory issues, weakness, and gait instability post-surgery (Gallay et al., 2020; Sinai et al., 2020; Abe et al., 2021; Agrawal et al., 2021; Lu et al., 2022).

### 3.8 Co-citation analysis

**Figure 9:**
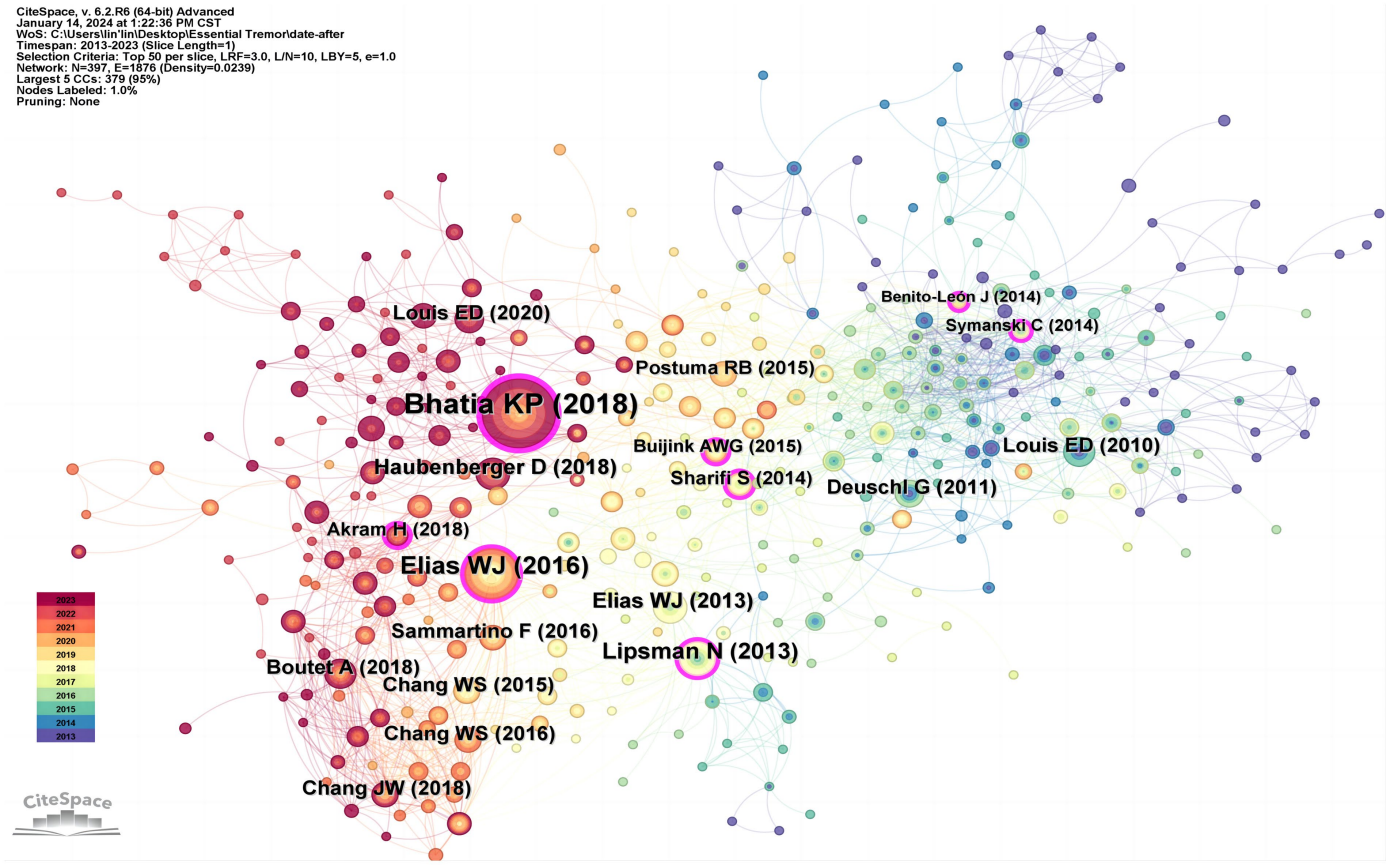
Highly-cited publications and co-cited references.

In instances where two or more papers are referenced by another publication, a co-citation relationship is established among these referenced works in the academic literature. This phenomenon of co-citation is visually represented through a network map, where ‘references’ serve as nodes, culminating in a total of 613 nodes (Figure 9).

The primary metric employed in this analysis is the frequency of co-citations, as outlined in Table 6. Citation frequency stands as a crucial metric for assessing the significance of academic literature, reflecting its scholarly impact within the corresponding field. The top 10 co-cited publications predominantly consist of 1 consensus paper (Bhatia et al., 2018), 3 review articles (Louis and Ferreira, 2010; Haubenberger and Hallett, 2018; Louis and Faust, 2020a), 2 Cross-S studies (Boutet et al., 2018; Chang et al., 2018), 1 RCT (Elias et al., 2016), and 3 SAT papers (Elias et al., 2013; Lipsman et al., 2013; Chang et al., 2015).

**Table 6:** The top 10 highly cited studies.

Rank	Title	Type	Year	Centrality	Journal	IF
1	Consensus Statement on the Classification of Tremors. From the Task Force on Tremor of the International Parkinson and Movement Disorder Society	Consensus	2018	0.23	Movement Disorders	8.5
2	A Randomized Trial of Focused Ultrasound Thalamotomy for Essential Tremor	RCT	2016	0.15	New England Journal of Medicine	158.5
3	MR-guided focused ultrasound thalamotomy for essential tremor: a proof-of-concept study	SAT	2013	0.12	Lancet Neurology	48
4	A Pilot Study of Focused Ultrasound Thalamotomy for Essential Tremor	SAT	2013	0.06	New England Journal of Medicine	158.5
5	Essential Tremor	Review	2018	0.04	New England Journal of Medicine	158.5
6	How Common Is the Most Common Adult Movement Disorder? Update on the Worldwide Prevalence of Essential Tremor	Review	2010	0.06	Movement Disorders	8.6
7	A Prospective Trial of Magnetic Resonance-Guided Focused Ultrasound Thalamotomy for Essential Tremor: Results at the 2-Year Follow-up	Cross-S	2018	0.03	Annals of Neurology	11.2
8	Unilateral magnetic resonance guided focused ultrasound thalamotomy for essential tremor: practices and clinicoradiological outcomes	SAT	2015	0.01	Journal of Neurology Neurosurgery And Psychiatry	11.8
9	Focused ultrasound thalamotomy location determines clinical benefits in patients with essential tremor	Cross-S	2018	0.03	Brain	14.5
10	Essential tremor pathology: neurodegeneration and reorganization of neuronal connections	Review	2020	0.03	Nature Reviews Neurology	38.1

These top 10 most frequently cited references reveal the pivotal themes dominating essential tremor research over the last decade. An analysis of the literature’s content shows a predominant focus on surgical treatments among researchers. Within this group of papers, 6 specifically address focused ultrasound thalamotomy, encompassing 1 RCT (Elias et al., 2016), 2 Cross-S studies (Boutet et al., 2018; Chang et al., 2018), and 3 SAT papers (Elias et al., 2013; Lipsman et al., 2013; Chang et al., 2015). This demonstrates the rising prominence of MRgFUS as a favored surgical intervention for essential tremor. Notably, Elias WJ published clinical trials on MRgFUS in the New England Journal of Medicine (IF = 158.5) in 2013 (Elias et al., 2013) and 2016 (Elias et al., 2016). The outcomes of these trials highlight the efficacy of MRgFUS in reducing hand tremor scores, ameliorating disability, and enhancing the quality of life for patients with essential tremor. However, it is also important to note the potential side effects, including sensory disturbances and gait disorders, associated with this treatment.

## 4 Discussion

This study employed the Web of Science (WOS) database to retrieve literature relevant to essential tremor spanning the years 2013 to 2023. Citespace 6.2.R6 software was then utilized for an in-depth analysis of the literature, aiming to identify current research hotspots and emerging frontiers in the study of essential tremor.

### 4.1 Current state of research

Over the past decade, research on essential tremor has been on the rise, with the highest volume of publications occurring from 2020 to 2022. Essential tremor is progressively emerging as a focal point of research interest. The United States leads in publication volume, with the top five contributing institutions all based in the United States, thus establishing a dominant position in this field. However, inter-institutional collaborations appear to be limited.

Keyword co-occurrence and clustering analysis indicate，recent trends suggest research on ET has predominantly concentrated on epidemiology, pathophysiology, and treatment. Notably, treatment studies have garnered the most attention including advanced surgical techniques like DBS and MR-guided focused ultrasound thalamotomy. Analysis of keyword bursts reveals, significant shifts in the prevailing topics of ET research have occurred over the past decade. Ten years ago, burst keywords suggested that ET research primarily centered on genetics, symptoms, risk factors, and pathogenesis. At this stage, international understanding of ET was still in its exploratory stage. Subsequently, the focus of research gradually shifted to treatment, with medications and surgical interventions becoming mainstream areas of study. Presently, research on ET has progressed beyond the initial understanding of the disease to explore its mechanistic underpinnings more deeply. Technological advancements have also paved the way for advancements in surgical procedures.

In the visualization analysis conducted Elan D. Louis from the University of Texas Southwestern Medical Center stands out with the highest number of publications (69 papers) focusing on the pathomechanism of essential tremor. Louis’s work includes a review of previous studies that link essential tremor to cerebellar dysfunction suggesting that essential tremor may be the most prevalent form of cerebellar degeneration (Louis, 2016a,b; Louis and Faust, 2020b,c).

Regarding journals, Movement Disorders is distinguished by leading in both publication volume and citation count. In the last decade, Movement Disorders published 581 articles on essential tremor, representing 1/4 of the total publications in this field. The most frequently co-cited paper is the “Consensus Statement on the Classification of Tremors from the Task Force on Tremor of the International Parkinson and Movement Disorder Society,” also featured in Movement Disorders. This underscores the pivotal role of the Movement Disorders journal in the realm of essential tremor research.

### 4.2 Research hotspots and frontiers

The visualization research results, including keyword co-occurrence, burst analysis, and co-citation clustering, effectively reveal the evolving research trends and focal points in essential tremor. The findings of bibliometric analysis reveal a substantial increase in academic publications over the past decade pertaining to the biological mechanisms, epidemiology, genetics, and treatment modalities of ET.

The incidence rates of ET vary geographically, with differences observed among countries and regions. In 2021, Louis and McCreary conducted an extensive review of the most recent epidemiological data related to ET. Their comprehensive analysis integrated findings from 42 studies conducted in 23 countries across six continents, revealing a global prevalence of ET at 1.33%, showing an association with age. Surprisingly, the study discounted any correlation between ET and gender. However, the amalgamated literature in this study exhibited significant heterogeneity, potentially compromising the reliability of the outcomes. Notably, an investigation into the prevalence of pediatric ET reported an incidence rate of 0.41%.

The genetics of ET remains a longstanding and significant topic within the field. Research on genetic testing for ET is crucial and has the potential to enhance our understanding of the pathophysiology of ET. The growing body of literature in recent years reflects an increasing interest in this area. Reports indicate that 30–70% of individuals with ET have a positive family history (Wagle, 2022). So far, genetic association studies have pinpointed a minimum of 20 genes that may confer either increased protection or heightened risk for developing ET. However, current genetic studies often yield conflicting results, potentially due to ethnic disparities, necessitating further research for validation (Deng et al., 2019; Siokas et al., 2020).

The investigation into the pathophysiological mechanisms of ET remains at the forefront of research. The specific mechanisms driving tremor generation in ET are still not fully elucidated. Currently, prominent hypotheses include the neurodegenerative, central oscillatory network, and GABAergic hypotheses (Helmich et al., 2013). Recent bibliometric findings indicate an increasing involvement of the cerebellum in the pathophysiology of primary tremor (Benito-León and Labiano-Fontcuberta, 2016; Marin-Lahoz and Gironell, 2016; Louis, 2018). Postmortem analysis of ET patients’ brains has revealed cerebellar damage, with significant pathological alterations in Purkinje fibers, such as Purkinje cell loss and axonal swelling. Neurons in proximity to Purkinje fibers may also be impacted (Louis and Faust, 2020a; Van den Berg and Helmich, 2021; Louis et al., 2023). Alongside cerebellar morphological changes, research is delving into molecular pathways encompassing calcium signaling, synaptic transmission, axonal guidance, microtubule activity, and intracellular transport pathways between the endoplasmic reticulum and the Golgi apparatus (Louis and Faust, 2020a; Van den Berg and Helmich, 2021). Furthermore, the locus coeruleus (LC) represents a promising target. The recent implementation of neuromelanin-sensitive MRI (NM-MRI) techniques has furnished quantitative tools for assessing the LC and substantia nigra (SN) (Shill et al., 2008; Chen et al., 2014). Research has identified marked LC degeneration in individuals with ET (Wang et al., 2023). Additionally, Lv et al. (2023) noted a heightened severity of LC degeneration in non-head tremor subtype of essential tremor.

With the advancement of technology and further research into the pathophysiological mechanisms, surgical techniques for ET have been rapidly updated. Deep brain stimulation (DBS) has recently overtaken thalamotomy as the preferred surgical approach for treating essential tremor (Kiss et al., 2003). Conventionally, DBS has targeted the ventral intermediate nucleus (ViM) of the thalamus, involving the placement of unilateral or bilateral leads (Diaz et al., 2020; Wong et al., 2020). The efficacy of this method has been well-established through extensive clinical use (Ferreira et al., 2019; Krauss et al., 2021). However, the posterior subthalamic area (PSA) has gained attention as an alternative target (Iorio-Morin et al., 2020). A meta-analysis by Fan et al. (2022) reviewed recent DBS studies targeting both ViM and PSA, revealing that PSA is superior to ViM in terms of tremor improvement and sensitivity, and has fewer stimulation-related complications (SRC). Nonetheless, the incidence of common DBS complications, such as dysarthria and gait ataxia, does not significantly differ between the two targets. It is noteworthy that previous studies on the ViM target were primarily small-scale or single-sample, potentially affecting their reliability. Therefore, the asserted superiority of ViM necessitates further validation through large-scale, multi-center randomized controlled trials”.

In addition, transcranial magnetic resonance-guided focused ultrasound (MRgFUS) has emerged as a promising surgical technique. This innovative method allows the focused ultrasound to penetrate the skull without heating the bone (van der Stouwe et al., 2023). The VIM nucleus of thalamus is a typical ablation target (Su et al., 2020; Jameel et al., 2022). The non-invasive nature of transcranial MRgFUS significantly reduces surgical risks (Ferreira Felloni Borges et al., 2023). However, it’s important to note that the current efficacy of MRgFUS does not seem to be satisfactory. In studies to date, unilateral MRgFUS thalamectomy in patients with essential tremor resulted in only about 40% improvement on the Clinical Rating Scale for tremor (CRST) at 3 months after treatment (Elias et al., 2016), which is less than the improvements observed with traditional deep brain stimulation (DBS) and radiofrequency thalamotomy. Additionally, side effects such as sensory, motor, and gait abnormalities can occur in up to 40% of MRgFUS thalamotomy cases (Germann et al., 2024). Interestingly, the efficacy of MRgFUS also appears to be related to the target of action (Jameel et al., 2022). Research by Alexandre and colleagues suggests that the effectiveness and acute adverse reactions of MRgFUS in essential tremor patients highly depend on the precise location and size of the focused ultrasound thalamotomy. A cross-sectional study (Germann et al., 2024) using diffusion-weighted imaging after MRgFUS surgery identified lesions associated with sensory abnormalities, gait/discrimination impairments, motor disorders, and speech abnormalities, affecting the medial lemniscus, dentato-rubro-thalamic tract, and corticospinal tract. Jurgen and colleagues (Germann et al., 2024) also confirmed that variations in the location of MRgFUS lesions influence the rate of adverse reactions. However, the identification of optimal targeting locations for better efficacy and fewer adverse reactions requires further research.

In movement disorders, the use of botulinum toxin is relatively widespread, as evidenced in conditions like Parkinson’s disease, multiple sclerosis, and essential tremor (Niemann and Jankovic, 2018; Zheng et al., 2020; Samotus et al., 2022). Botulinum toxin works by inhibiting acetylcholine release at neuromuscular junctions, thus blocking nerve impulses between nerves and muscles and effectively reducing tremors (Liao et al., 2022; Marques et al., 2023). However, when used in essential tremor, it may lead to muscle weakness at the injection site. Recently, principles of precision medicine have been applied to botulinum toxin treatments. The Yale protocol (Mittal et al., 2017) and computerized kinematic tremor assessment (Samotus et al., 2017) have substantially improved the management of side effects. The Yale protocol involves pre-injection electromyography screening of 8–10 forearm muscles, including two proximal and lumbar muscles, to plan the treatment strategy. The computerized kinematic tremor assessment uses multi-sensor kinematic technology on the entire arm to identify tremor-affected muscle groups, enabling the customization of BoNT injections for 7 forearm and 6 proximal muscles. Studies (Samotus et al., 2017; Mittal et al., 2020) have shown that customized toxin dosing offers superior treatment effectiveness and reduced side effects compared to fixed-dose, fixed-muscle injections. However, there are currently no updated protocols for botulinum toxin injections targeting head tremors. In a study by Ana and colleagues (Marques et al., 2023) involving injections into the sternocleidomastoid muscle, a significant effect was observed in 31% of patients, but over 50% experienced side effects such as neck pain, muscle weakness, and swallowing difficulties.

## 5 Conclusion

In a groundbreaking initiative, this study utilizes Citespace for an extensive visual analysis of the literature on essential tremor. It offers a clear and intuitive overview of the current research landscape, highlighting key areas of focus and potential future development trends in the field. The notable research areas and emerging trends identified include: firstly, analyzing the global epidemiology of ET to determine its prevalence across diverse regions and age groups; secondly, conducting genetic testing to pinpoint genetic loci linked to ET and investigate interethnic variations; thirdly, delving into the pathophysiological mechanisms of essential tremor, placing particular emphasis on the correlation between cerebellar function and molecular underpinnings; fourthly, exploring alternative surgical strategies for treating essential tremor and identifying optimal targets for DBS and MRgFUS; and lastly, advancing the treatment of essential tremor through innovations in botulinum toxin injections.

## Author contributions

LZ: Writing – original draft. SC: Writing – review & editing. XX: Conceptualization, Writing – original draft. HB: Software, Writing – review & editing. BH: Writing – review & editing.
